# Effects of salinity stress on morphological structure, physiology, and mRNA expression in different wheat (*Triticum aestivum* L.) cultivars

**DOI:** 10.3389/fgene.2025.1535610

**Published:** 2025-05-30

**Authors:** Xiaohui Sun, Yuliu Tan, Yumei Zhang, Weiwei Guo, Ximei Li, Nataliia Golub, Lili Zhang, Huifang Wang

**Affiliations:** ^1^ Shandong Engineering Research Center of Germplasm Innovation and Utilization of Salt-Tolerant Crops, Qingdao Agricultural University, Qingdao, Shandong, China; ^2^ Agricultural Biotechnology Research Institute, Yantai Academy of Agricultural Sciences, Yantai, Shandong, China; ^3^ Academy of Dongying Efficient Agricultural Technology and Industry on Saline and Alkaline Land in Collaboration with Qingdao Agricultural University, Qingdao Agricultural University, Dongying, Shandong, China; ^4^ Environmental Biotechnology and Bioenergy Department, Igor Sikorsky KPI, Peremohy 37, corpus 4, Kyiv, Ukraine

**Keywords:** wheat, salt stress, morphology, physiology, structure, gene expression

## Abstract

Salinity is a major abiotic stress that threatens crop yield and food supply in saline soil areas. Wheat (*Triticum aestivum* L.) is the most important cereal crop in arid and semiarid land areas, which are often adversely affected by soil salinity. Hence, creating salt tolerance wheat is of great value for utilizing saline soils. In this study, two wheat cultivars QingMai 6 (QM6, salt-tolerant) and Chinese Spring (CS, salt-sensitive) were subjected to salinity stress. Morphological analysis showed that the seedlings of QM6 grew better than CS under salt stress conditions, especially in roots. Electron microscopic studies revealed that salinity stress caused significantly more root hairs and less effect on normal chloroplast structure in QM6 than these in CS. Moreover, QM6 showed a higher photosynthetic activity under salt stress conditions compared to CS. Further investigation showed the salt-tolerant phenotypes of QM6 were accompanied by decreases of reactive oxygen species (ROS) content, and lower antioxidant enzyme activities after salt treatment compared with CS. Additionally, qRT-PCR analyses revealed that the expression level of ROS-scavenging genes (*TaSOD6*, *TaCAT1/5/6*, *TaPOD7, TaP5CS1*) and stress-responsive genes (*TaDREB3*, *TaWRKY19*, *TaERF5a, TaLTP1, TaTIP2*) displayed more transcripts in QM6 than CS. These results provide insight into the mechanisms underlying salt tolerance in wheat, and could be potentially used to develop salt tolerant wheat varieties.

## 1 Introduction

Currently, soil salinization affects more than 20% of all cultivated land and approximately half of all irrigated land in the word, which will probably increase in extent and severity due to seawater intrusion and intensive irrigation practices aggravated global climate change (Hu and Schmidhalter, 2023; Hu et al., 2025) Soil salinity can harm all stages of plant growth from seed germination to reproductive stage, leading to substantial crop yield losses (Shannon, 1997; Pessarakli and Kopec, 2009). Indeed, in 2021, the global annual cost of salt-induced land degradation in irrigated areas was estimated to be US$27.3 billion related to lost crop productivity by the Food and Agriculture Organization of the United Nations (Colin et al., 2023). Therefore, it is necessary to improve the salt tolerant of modern germplasms and cultivars. This requires a comprehensive understanding of the salt tolerance mechanism in crops (Colin et al., 2023; Yang et al., 2023).

Salinity caused osmotic stress, ionic toxicity, and secondary stresses, particularly oxidative stress (Yang and Guo, 2018), together with various morphological, physiological, biochemical, and molecular changes in plants, which directly inhibit plant growth and development (Munns and Tester, 2008; Hu and Schmidhalter, 2023; Zelm et al., 2020; Hu et al., 2025). In the short-term, osmotic stress is actually caused by water deficit and inhibits the growth of new shoots and young leaves, due to the increase of salt concentration (NaCl) to a threshold level. Osmotic stress influences various aspects of plant physiology, including water absorption, seed germination, cell elongation, leaf development, lateral branching, photosynthesis rate, nutrient uptake and translocation from root to shoot, abrupt supply of carbohydrates to meristematic tissues, and ultimately exerts a negative impact on overall growth of the plant (Munns and Tester, 2008; Zelm et al., 2020). Then, at long time high salt concentration in in plant cells further generates ion toxicity. Ion toxicity of Na^+^ and Cl^−^ disrupts the uptake of essential nutrients such as Ca^+^ and K^+^, resulting in a nutritional imbalance within plants (Acosta-Motos et al., 2017).Moreover, when an excessive amount of salt ions get into the transpiration stream of plants, they cause harm to plant cells by inhibiting photosynthesis and impairing ion homeostasis (Barba-Espín et al., 2011), thus further negatively affecting plant vegetative and reproductive growth. Oxidative stress is caused by primary stresses (osmotic stress and ion toxicity) under salt stress, due to the excess production of reactive oxygen species (ROS) during metabolism. Excessive ROS synthesis can cause cell damage, such as lipid peroxidation, RNA and DNA molecules, proteins and debilitation of the metabolic processes (Mittler, 2002; Garcia de la Garma et al., 2014; Yang and Guo, 2018). Overall, salinity is responsible for different types of stresses, mainly osmotic stress, ionic stress, and oxidative stress, which together obstruct physiological and biochemical activities of the plant, and result in impairing plant growth and development.

Plants as sessile organisms cannot simply move and must cope with salt stress (Zhu, 2016). To adapt to salt stress, plants have evolved various strategies to optimize the balance between growth and stress response. The main of tolerance mechanisms is activated to ensure the maintenance of ion homeostasis in plant cells, through regulating the balance between potassium (Jadidi et al., 2022). Maintaining an optimal photosynthesis process constitutes another pivotal mechanism for achieving a high level of tolerance. Reduction in photosynthetic pigment contents and photosynthesis rate under salt stress have been used as the key biochemical and physiological indicator to screen salt-tolerant genotypes, respectively (Parihar et al., 2014). Additionally, salt treatment also induces the generation of ROS in plant cells. ROS are mainly comprised of free radicals such as O_2_^−^ (superoxide radical), and H_2_O_2_ (hydrogen peroxide) (Kaushik and Aryadeep, 2014). Plants have developed a multifaceted antioxidant defense mechanism to detoxify excess ROS accumulates under salt stresses. The antioxidant defense system consists of two different types of antioxidants, namely, antioxidant enzymes (SOD, superoxide dismutase; CAT, catalase; POD, peroxidase; APX, ascorbate peroxidase, and so on) and non-enzymatic antioxidants (AsA, ascorbic acid; GSH, glutathione; CAR, carotenoids, and so on) (Gupta and Huang, 2014; Azeem et al., 2023). Increasing evidence indicates that salt stress-inducible molecular processes including numerous salt-responsive genes and transcription factors (TF) intersect with each other and form a complex network to regulate the physiological response of plants to salt stress. Ca^2+^ including two categories acts as an important pathway, which are a Ca^2+^-dependent signal transduction pathway (SOS pathway, ABA pathway, CDPK pathway) and a Ca^2+^-independent signal transduction pathway (MAPK pathway) (Hao et al., 2021; White and Broadley, 2003). All in all, there is a complex network of physiological and molecular mechanisms involved in salinity tolerance. Hence, the development of new superior salt-tolerance varieties needs a comprehensive investigation of various growth aspects and molecular (Mittler, 2002).

Wheat (*Triticum aestivum* L.) is one of the most important cereal crops widely planted all over the world, which is a staple food for more than one-third of the world’s people (Shiferaw et al., 2013). Considering the limited arable land area, increasing world’s population, urbanization, environmental changing, and COVID-19, wheat production cannot meet the demand of global population without a new agricultural revolution. To increase the wheat production, especially in salt affected arid and semiarid areas, creating salt-tolerant wheat cultivars is of great value. Wheat is also a moderately salt-tolerant crop that is more tolerant than rice (*Oryza sativa*) but less tolerant than barley (*Hordeum vulgare*) (Munns and Tester, 2008). It has been reported that salinity has inhibitory effects on wheat root and shoot traits, even ultimately reduces wheat production (Asgari et al., 2012). Wheat salt tolerance is a complex trait that is controlled by multiple genes and involves various biochemical and physiological mechanisms (Zhang and Shi, 2013). The performance of the morphological, physiological, biochemical parameters, and gene regulation will vary among wheat genotypes, with a genotype being superior in at least one trait and inferior in other traits under salt stress. Thus, genetic variations in salt tolerance exist and the degree of salt tolerance varies in different wheat genotypes (Naz et al., 2015). Although much has been documented about negative impacts of salt stress in wheat, its effects on morphological, physiological, biochemical, cellular and molecular mechanisms regulating plant adaptation and tolerance to salinity stress in different wheat genotypes are largely unknown (Hayat et al., 2021). In the present study, we aimed to explore the effects of salinity stress on some morphological, physiological, biochemical and cellular characteristics of wheat and also to elucidate the salt tolerance mechanism of wheat genotypes. Hence, we used a salt-tolerant wheat cultivar (QM6) and a salt-sensitive cultivar (CS) to demonstrate the different responses and adaptations to salt stress at morphological, structural, physiological, biochemical, and molecular levels. The identification of salt-tolerant mechanism in salt-tolerance wheat cultivars could represent valuable resources for genetic improvement programs to provide greater understanding of plant tolerance to salt stress, supporting agricultural production on salinized soils.

## 2 Materials and methods

### 2.1 Plant materials and growth conditions

Two bread wheat cultivars were selected for this study, including a spring wheat variety Chinese Spring (CS) and a winter wheat variety Qingmai 6 (QM6). CS was used as a salt-sensitive cultivar, and QM6 was used as a salt-tolerant cultivar. Wheat seeds were uniformly selected and sterilized with 2% NaClO for 20 min. The soaked seeds were placed in Petri dishes lined with filter paper and soaked with sterile water. After this, the seeds were incubated at 4°C for 3 days in the dark and then germinated at room temperature for 2 days. Uniformly germinated seeds were grown in greenhouse with a 16-h/8-h light/dark photoperiod, a temperature regime of 24:18°C (light: dark), a light intensity of 14,100 lx, and 55% humidity. The 2-day-old seedlings were transplanted into the culture boxes with 1/8 Hoagland’s solution for 8 days in the greenhouse (Hoagland and Arnon, 2018). The 10-day-old uniform seedlings were treated with various concentrations of NaCl (0%, 0.15%, 0.30%, 0.45% and 0.60%; w/w, dry soil weight base) and 1/8th strength Hoagland solution. The solution was renewed every 3 days during the growth of the wheat seedlings.

### 2.2 Measurements of root length, root surface area, shoot length and leaf surface area

The 10-day-old seedlings were treated with different salt solutions for 10 days, and then separated out their roots and leaves, respectively. The root surface area and leaf surface area were measured with a scanner (EPSON PERFECTION V700 PHOTO), respectively. The data was analyzed by EPSON Scan Software. The root length and shoot length was measured with a ruler, respectively. Each genotype of each treatment was performed with three biological replicates, and the number of seedlings for each replicate was at least 8.

### 2.3 Scanning electron microscope (SEM) observation of root hairs

For root hair epidermal observation, the mature zone of both control roots and those treated with 0.3% salt solution for 10 days were fixed in FAA. After dehydrated in ethanol and dried at critical point, they were coated with gold in the vacuum evaporator. SEM was performed as described previously (Vila et al., 2005). The microstructure of roots under control and salt conditions was performed by using a HITACHI S-3400N scanning electron microscope (Hitachi, Tokyo).

### 2.4 Transmission electron microscopy (TEM) observation of chloroplast structure

After treated with 0.3% salt solution for 10 days, 1–2 mm^2^ small pieces of leaves of wheat seedlings were fixed in 2.5% glutaraldehyde. They were washed by 0.1M phosphate buffer and fixed in 1% osmium tetroxide, dehydrated in ethanol and acetone, and then embedded in resin. Ultrathin section (100 nm) leaf specimens were prepared using a Leica ultra microtome EM UC7 (Leica, Austria), and the specimens were mounted on copper grids. The specimens were stained with 5% aqueous uranyl acetate for 15 min and Reynold’s lead citrate for 15 min and examined under a transmission electron microscope (HT7700, HITACHI, Japan).

### 2.5 Measurements of photosynthesis index

The LI-6800 portable photosynthesis analyzer (LI-COR, Lincoln, NE, United States) was used to measure the intercellular CO_2_ concentration (Ci), transpiration rate (Tr), stomatal conductance (Gs) and net photosynthetic rate (Pn). The measurements were conducted as described by Tian (Tian et al., 2019).

### 2.6 Measurements of physiological-biochemical parameters

Seven-day-old CS and QM6 seedlings were treated by 0.30% NaCl were subjected to normal and salt treatments for 7 days. Leaves were then collected for determination of physiological-biochemical parameters in CS and QM6 under control and salt stress conditions. The soluble sugar content (G0501W/20240311, Grace Biotechnology, Suzhou, China), proline content (G0111W/G182411131R01/02, Grace Biotechnology, Suzhou, China), SOD activity (G0101W/G020240920R01/02, Grace Biotechnology, Suzhou, China), POD activity (G0107W48/20240304, Grace Biotechnology, Suzhou, China), O_2_^−^ (G0116W48/20240507, Grace Biotechnology, Suzhou, China) and H_2_O_2_ (G0168W/G182410251R01/02, Grace Biotechnology, Suzhou, China) content were determined using corresponding reagent kits following manufacturer’s instructions. All measurements were performed in triplicate, with a sample weight of 0.1g each time.

### 2.7 RNA extraction and reverse transcription qPCR (RT-qPCR) analysis

Salt stress was applied to the 10-day seedlings of QM6 and CS by the addition of 0.3% NaCl solution to the hydroponic solution. Leaves were collected after stress treatment for 6 h and frozen immediately in the liquid nitrogen for further use. Total RNA was extracted using the EASY spin reagent following the manufacturer’s instructions. DNA contamination was removed with DNaseⅠ (RNase-Free). First strand cDNA was synthesized from 2 μg total RNA using the PrimerScript™ RT reagent Kit (TaKaRa, Dalian, China) according to the manufacturer’s instructions. RT-qPCR was conducted using ChamQ Universal SYBR qPCR Master Mix (Vazyme, Nanjing, China) with a Miniopticon Real-Time PCR instrument (ABI2700). The RT-qPCR conditions and analytical methods were the same as those described by (Wang et al., 2018). The specific gene primers for qPCR were designed according to the conserved region of three homeologs. The wheat Actin gene was used as a universal reference gene. The accession numbers of *TaActin* homoeologous genes were *TraesCS1A02G274400*, *TraesCS1B02G283900*, and *TraesCS1D02G274400* (Shimada et al., 2009). Each sample was quantified in triplicate. A description of the genes and primer sequences was given in Supplementary Table S1.

### 2.8 Statistical analysis

Data shown in the figures for quantification analyses were presented as mean values ±SD. Student’s t-test and Duncan’s test was conducted using IBM SPSS Statistics 26.0 (SPSS, Chicago, United States). All statistical tests were performed by two-sided significance tests with a 0.05 and 0.01 significance level.

## 3 Results

### 3.1 Effect of salinity on wheat growth

**FIGURE 1:**
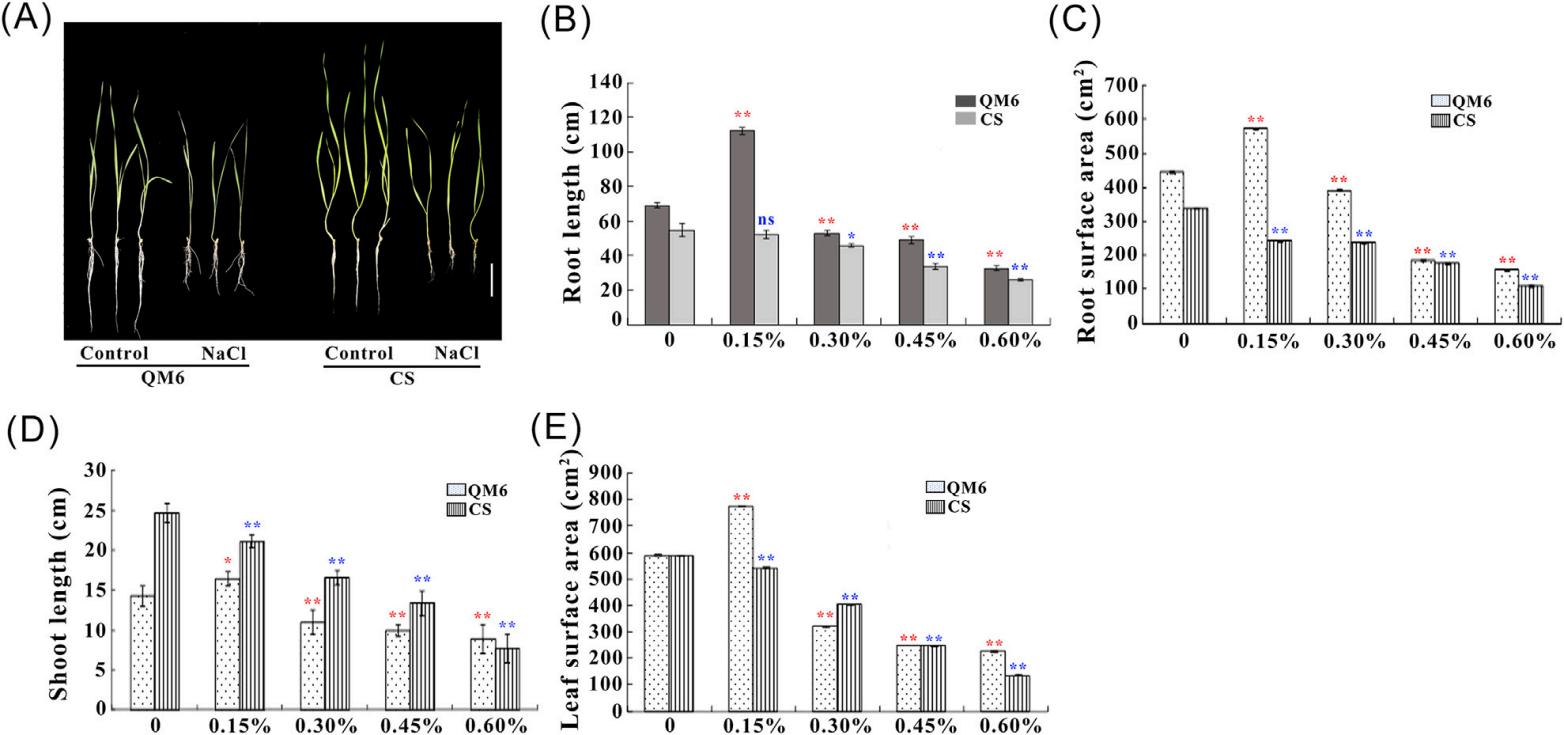
Effect of different salinity treatments on root and shoot growth in the salt-sensitive cultivar (CS) and salt-tolerant cultivar (QM6) **(A–D)**. **(A)** Phenotype of 10-day-old QM6 and CS under control or 0.30% NaCl. **(B)** root length. **(C)** root surface area. **(D)** shoot length. **(E)** leaf surface area. All values are presented as mean ± SD. Statistical differences between control and salt treatment groups are indicated by asterisks and were determined using Student’s t-test: ns, not significant; *P < 0.05; **P < 0.01.

The present results showed that salt stress significantly affected the root and shoot growth of QM6 and CS (Figure 1). As shown in Figure 1A, wheat seedlings of QM6 grew better than those of CS under salt stress conditions, especially in roots. The root length of QM6 was significantly longer at 0.15% NaCl treatment and lower at different concentrations of NaCl (0.3%–0.6%) than that under control conditions, respectively (Figure 1B). Notably, QM6 had the longest root length when NaCl at 0.15% NaCl, whereas the root length of CS was not significantly different from the control when NaCl was applied at 0.15% NaCl. With increasing salt concentration (0.3%–0.6%), the root length of CS was reduced. The reduction of root length was substantially smaller in QM6 than that in CS (Figure 1B).

The root surface area of CS significantly decreased after NaCl treatment (0%–0.6%) (Figure 1C). The 0.15% NaCl treatment significantly increased the root surface area of QM6, but other NaCl treatments showed a significant decrease in root surface area compared with without treatment (Figure 1C). QM6 had larger root surface area at different salt concentrations (0, 0.15, 0.3, and 0.6%) compared with CS, while the root surface area of CS was similar with that of QM6 at 0.45% NaCl treatment (Figure 1C).

The NaCl treatment showed a significant decrease in shoot length of CS compared with the control (without treatment) at different salt concentrations (0.15%–0.60%) (Figure 1D). As shown in Figure 1C, the shoot length of QM6 slightly increased compared with the control when 0.15% NaCl was applied, but significantly reduced when 0.30% NaCl or above was applied. CS had longer shoot length than that of QM6 in response to different salt concentrations (0%–0.45%), whereas the shoot length of CS is not different from that of QM6 at 0.60% NaCl (Figure 1D). The observed reduction of shoot length in QM6 was significantly lower relative to that in CS (Figure 1D).

Compared to the control, the leaf surface area of both wheat cultivars was significantly decreased in response to salinity, except for QM6 at 0.15% NaCl (Figure 1E). There was a significant increase in leaf surface area of QM6 at 0.15% NaCl compared with the control (Figure 1E). As shown in Figure 1E, the leaf surface area of QM6 was similar with that of CS at 0% and 0.45% NaCl. Moreover, the reduction of leaf surface area was slightly higher in QM6 than CS at 0.30% NaCl, while lower reduction in QM6 in response to 0.60% NaCl (Figure 1E).

### 3.2 SEM analysis of wheat root hairs in response to salinity stress

**FIGURE 2:**
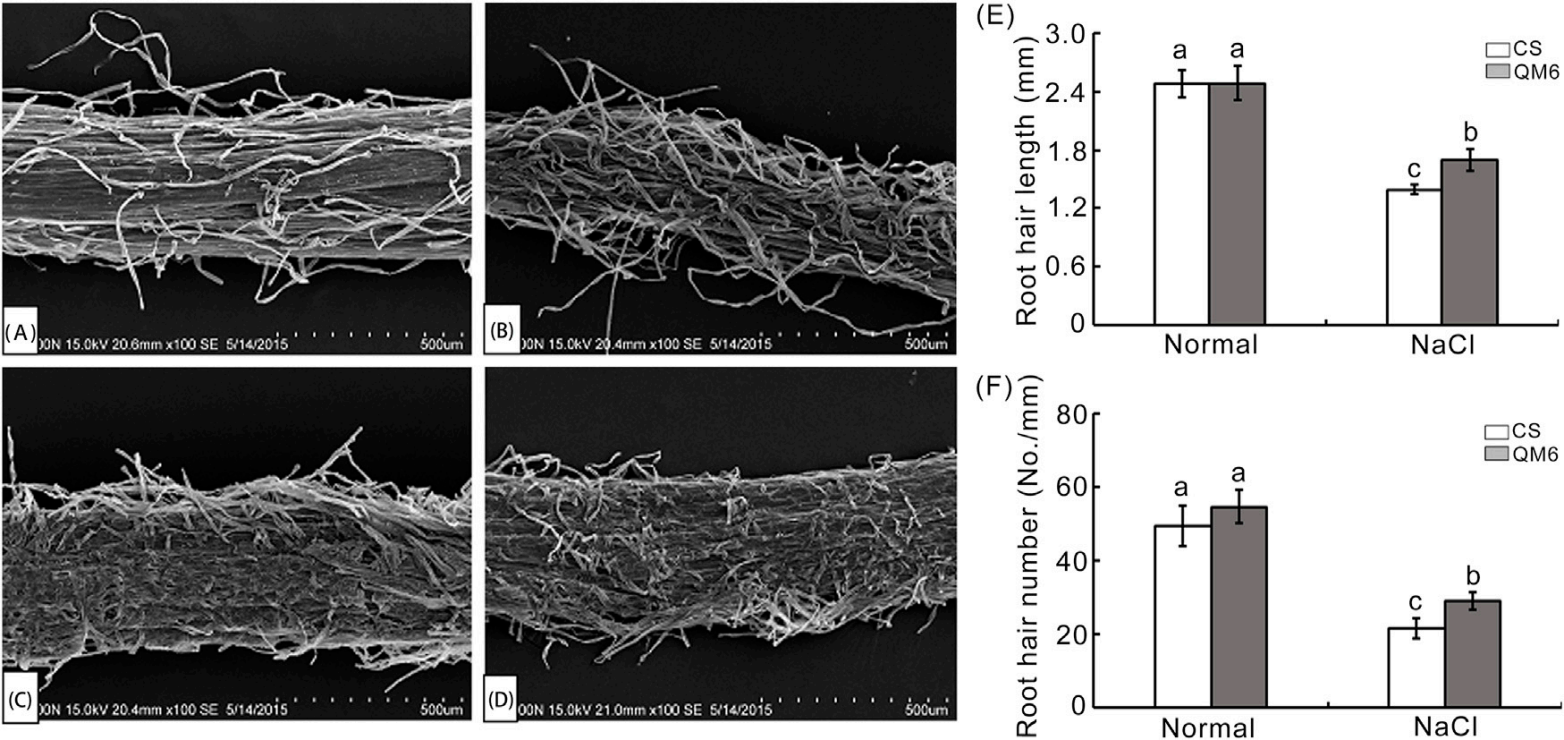
Scanning electron microscopy (SEM) of root hairs of the salt-tolerant cultivar (QM6) and salt-sensitive cultivar (CS) treated with 0.30% NaCl solution **(A–D)**. **(A, B)** SEM images of surface of root mature zone of QM6 under control **(A)** and 0.3% NaCl conditions **(B)**. **(C, D)** SEM photos of surface of root mature zone of CS under control **(C)** and 0.3% NaCl conditions **(D)**. **(E)** Quantitative analysis of root hair length. **(F)** Quantitative analysis of root hair number. The values are means ± SD of three biological replicates. The data were subjected to one-way ANOVA, and Duncan’s test at a 0 01 significance. Different letters are used to indicate means that differ significantly.

Root hairs are specialized plant cells and are responsible for greatly increasing root surface area making them important for water and ion uptake (Hussain et al., 2016; Bates and Lynch, 2000). The root hairs of QM6 and CS after 0.30% NaCl treatment were analyzed using a scanning electron microscope (SEM). As shown in Figure 2, there was no difference in root hair length and density between QM6 and CS under normal conditions (Figures 2A, C, E, F), whereas QM6 and CS both exhibited significantly decreased root hair length and density under NaCl treatment compared with the control (Figures 2B, D–F). In contrast, QM6 had much more and longer root hairs than CS under salt stress (Figures 2E, F).

### 3.3 TEM analysis of chloroplast structure in wheat mesophyll cell responding to salinity stress

**FIGURE 3:**
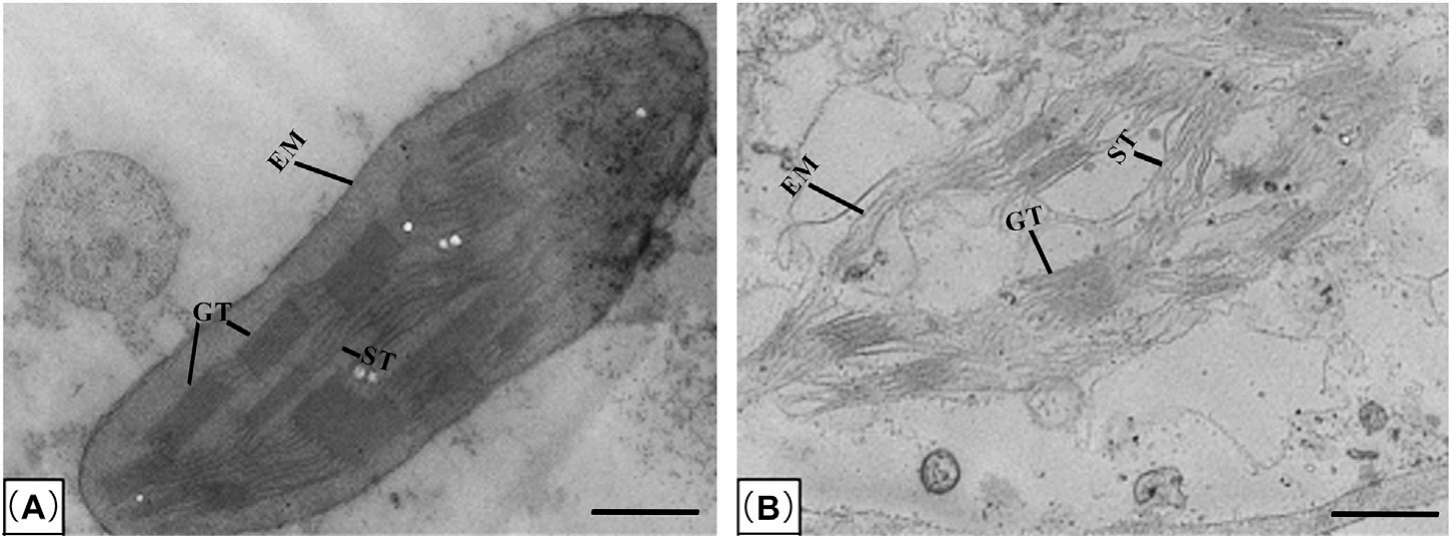
Transmission electron microscopy (TEM) of the chloroplasts in leaf mesophyll cells of salt-tolerant wheat variety QM6 **(A)** and salt-sensitive wheat variety CS **(B)** treated with 0.30% NaCl solution. EM, envelope membranes; GT, stacked grana thylakoids; ST, un-stacked stroma thylakoids. Bar: 1 μm.

Chloroplasts is highly integrated with cellular and plant development, which are oval and include double membrane, grana, stoma lamella and ground substance under the TEM (Yang et al., 2010). To determine the effect of salinity on the chloroplast structure in leaf mesophyll cell of wheat, TEM analysis was performed. As shown in Figure 3, after treated with 0.30% NaCl solution, the chloroplast shape of QM6 was oval. Moreover, it possessed clearly two envelope membranes, a well-developed internal membrane system, composed of grana and long stromal thylakoids (Figure 3A). Specifically, grana were distributed in a regular manner throughout the sectioned areas of chloroplasts, and granal thylakoids were well developed and packed closely together (Figure 3A). However, the chloroplast shape of CS was deformed and broken (Figure 3B). Its two envelope membranes were broken and incomplete (Figure 3B). Although the grana and stoma thylakoids of chloroplast could be seen, they were very irregular (Figure 3B).

### 3.4 Effects of salt stress on photosynthetic index in wheat

**FIGURE 4:**
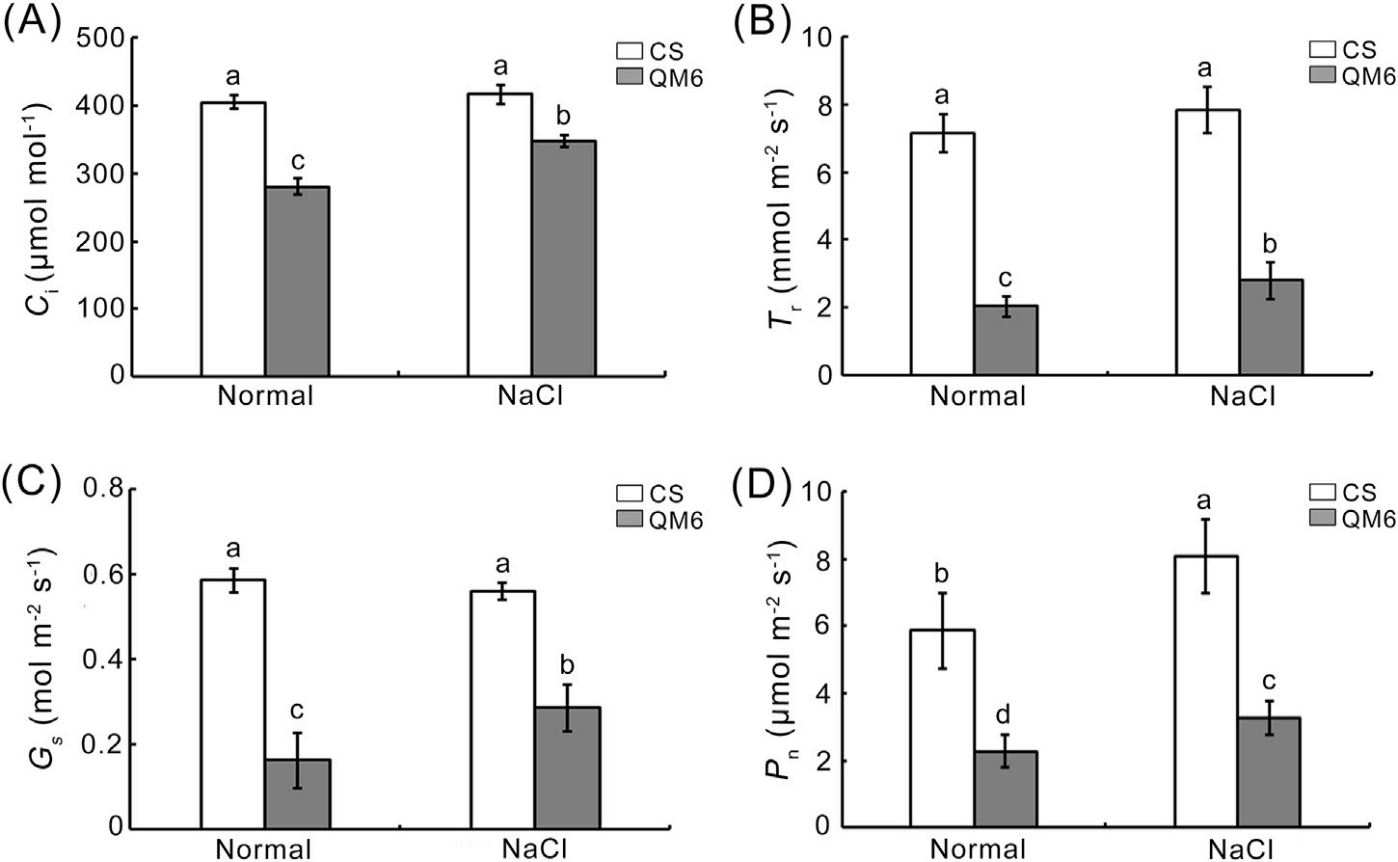
Effects of salt stress on photosynthetic index under salt stress. **(A)** internal CO2 concentration (Ci). **(B)** transpiration rate (Tr). **(C)** stomatal conductance (Gs) **(D)** Net photosynthetic rate (Pn). Different lowercase letters above the columns indicate significant differences between CS and QM6 under normal and salt stress conditions (Duncan’s test, p < 0.05).

Photosynthesis, together with cell growth, fuels a number of metabolic processes determining plant growth and yields, but it is substantially affected by salt stress (Feng et al., 2014). Our results showed that there was no significant difference in intercellular CO_2_ concentration (Ci), transpiration rate (Tr), and stomatal conductance (Gs) between CS and QM6 under normal growth conditions (Figures 4A–C), while net photosynthesis rate (Pn) was significantly higher in QM6 than CS under normal conditions (Figure 4D). Under salt stress, Ci, Tr, Gs, and Pn were significantly reduced for CS and QM6 response to NaCl, with larger reductions in CS (61.16%, 30.79%, 71.49% and 72.19%) than QM6 (16.51%, 64.23%, 48.87% and 59.44%) (Figure 4).

### 3.5 Effects of salt stress on physiological-biochemical parameters in wheat

**FIGURE 5:**
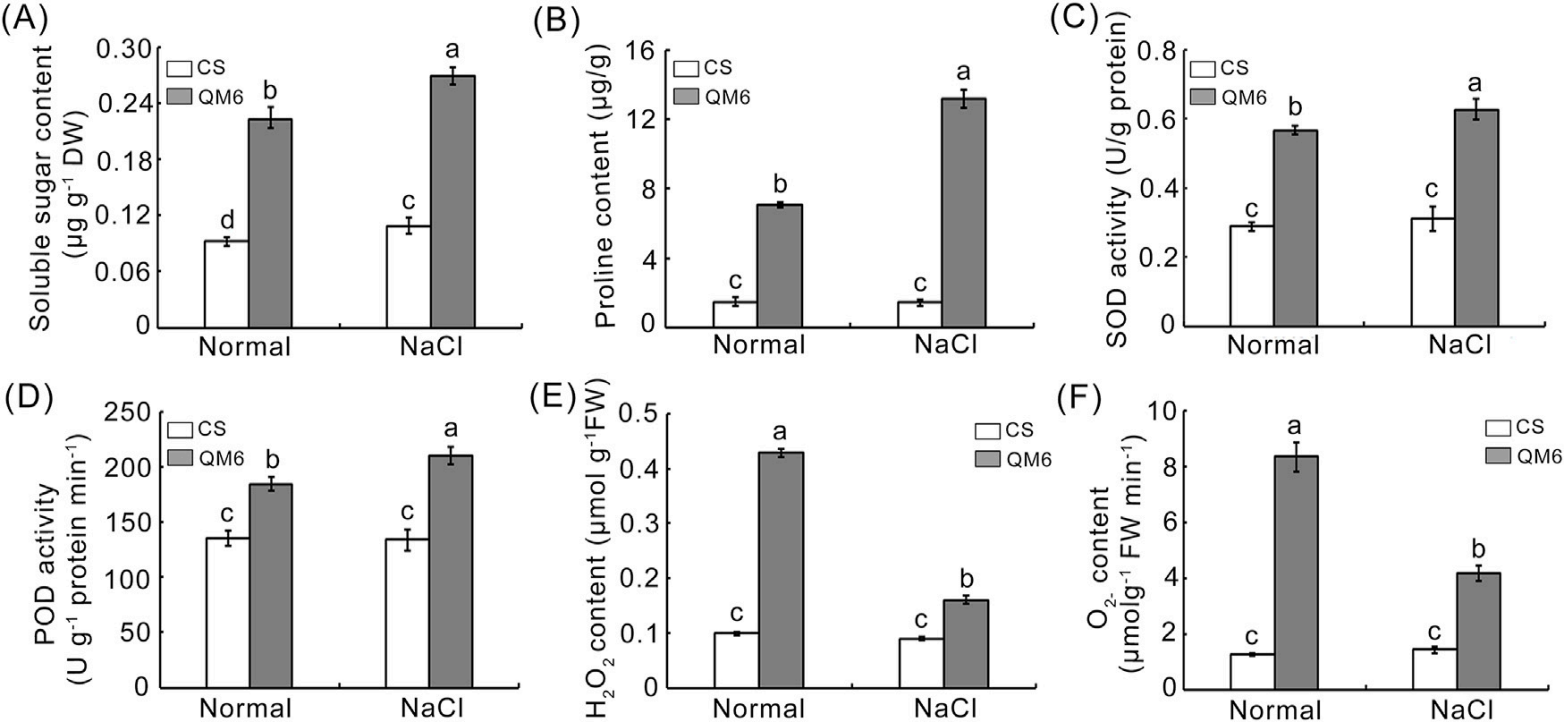
Determination of physiological traits in CS and QM6 under salt stress. **(A)** soluble sugar content. **(B)** proline content. **(C)** SOD activity. **(D)** POD activity. **(E)** H_2_O_2_ content. **(F)** O_2_^−^ content. The data presented are means ± SD of three biological replicates. Different lower-case letters above the columns indicate significant differences between CS and QM6 under normal and salt stress conditions (Duncan’s test, p < 0.05).

To uncover the potential physiological mechanisms underlying the different salt sensitivity between CS and QM6, we measured several physiological-biochemical parameters in CS and QM6 plants, including osmolytes (soluble sugar and proline), antioxidant enzyme activities (SOD and POD), and ROS accumulation (H_2_O_2_ and O_2_^−^). Under normal growth conditions, CS showed the same level of soluble sugar and a lower proline content as compared to QM6 (Figures 5A, B). However, after salt stress, both CS and QM6 exhibited increased soluble sugar and proline contents, but QM6 showed significantly higher soluble sugar and proline contents than those in CS (Figures 5A, B). Subsequently, the activities of SOD and POD were assessed in CS and QM6 plants. The results demonstrated that the activities of SOD and POD significantly increased after salt stress, and the increase in SOD and POD activities were less in CS plants than those in QM6 plants. In contrast, under normal conditions, there was no difference in SOD and POD between CS and QM6 (Figures 5C, D). In order to further understand the accumulation of ROS in the two varieties of wheat after salt treatment, the accumulation of H_2_O_2_ and O_2_^−^ was examined. Under normal growth conditions, there was no significant difference in the accumulation of H_2_O_2_ and O_2_^−^ between CS and QM6, whereas CS under salt stress showed more accumulation of H_2_O_2_ and O_2_^−^ than QM6 (Figures 5E, F). These results indicated that QM6 decreased ROS accumulation by increasing the activities of several antioxidant enzymes under drought stress compared to CS.

### 3.6 Expression analysis of stress-related genes in CS and QM6

Since the accumulation of ROS can directly damage different aspects of cell structure such as cellular membrane, lipids, proteins and nucleic acids (Hernández et al., 1995; Chawla et al., 2011), we wondered whether the expression patterns of ROS-scavenging genes and stress-responsive genes are affected. To test this idea, the transcript levels of ROS-scavenging genes (*SOD*, *CAT*, *POD* and *PSCS*) and stress-responsive genes (*TaDREB1*, *TaDREB3*, *TaERF5a*, *TaWRKY19*, *TaLTP1*, *TaAQP7*, and *TaTIP2*) were evaluated in CS and QM6 plants grown under salt treatment using qRT-PCR. Our results showed that the ROS-scavenging genes including *TaSOD1/6*, *TaCAT1/4/5/6*, *TaPOD7*, and *TaP5CS1/5* were upregulated in QM6 plants compared to the control under salt stress conditions. In contrast, *TaSOD6*, *TaCAT1/5/6*, *TaPOD7*, *TaP5CS1* were dramatically downregulated in CS plants compared to the control under salt stress conditions. There was no significant difference in the transcript levels of *TaSOD1* and *TaCAT4* in CS plants under normal growth conditions and salt stress conditions. Furthermore, the *TaP5CS5* gene expression were significantly enhanced in QM6 plants compared with CS (Figure 4B). Although the *TaDREB3*, *TaERF5a*, *TaWRKY19*, *TaLTP1*, and *TaTIP2* gene expressions were all upregulated in both QM6 and CS under salt stress conditions, the change in the degree of these upregulated genes in QM6 was significantly higher than that in CS. Moreover, the expression of *TaDREB1* and *TaAQP7* were significantly enhanced in QM6 plants whereas there was no significant change in CS under salt stress treatment (Figure 4B). Therefore, our results indicate that QM6 had stronger adaptability to salt stress than CS probably via inducing the expression of the downstream stress-related genes involved in ROS scavenging and defense mechanisms.

## 4 Discussion

Salinity is a foremost restriction to wheat production, especially in the world’s arid and semiarid regions (Hollington, 2000). Salt stress induces a series of negative effects in morphological and structure changes with increase in salinity levels (Saddiq et al., 2021). Considering that salt tolerance is a complex phenotype determined by multiple elite alleles controlling quantitative traits, different wheat varieties have different tolerance levels under salt stress conditions (Ami et al., 2020). Many researchers examined wheat genotypes for salt tolerance have been identified through morpho-physiological traits (Majeed et al., 2019; Mahboob et al., 2018; Hasanuzzaman et al., 2022). In the present study, a winter wheat genotype (QM6) and a summer wheat (CS) were carried out a comparative study on salt tolerance at morphological, structural and molecular levels. Overall, the results showed QM6 is better suited to grow on salinized soils. Therefore, QM6 could be utilized as valuable genetic resources in wheat breeding for salinity tolerance research.

Given that the seedling stage shows the most sensitive to salinity stress throughout the plant life cycle, the salt tolerance of seedling is very important to wheat production (Ma and Weng, 2005). Salinity has adverse impacts on water and mineral uptake which leads to reduction of seedling growth (Zheng et al., 2008). The wheat seedling growth performance significantly decreased in root and shoot growth under salt stress conditions, such as shoot height, root length, and dry weight of roots and shoots (Zheng et al., 2008). Moreover, in the present study, QM6 had well-developed root system and shoot growth compared with CS after salt stress (Figures 1A, B). After NaCl treatment, the root and shoot growth of QM6 was not inhibited under low salt concentration, but was increased compared to the control (without NaCl treatment). In contrast, there was an obvious reduction in root and shoot growth of CS under different salt concentrations compared with the control (without NaCl treatment) (Figure 1). Our root growth results are in accordance with the results of our previous study (Zhang et al., 2016). However, it has been reported that winter wheat had a lower root length than spring wheat under abiotic stress, due to spring wheat with a greater supply of assimilates from leaves to growing parts (Yang et al., 2010). However, different wheat varieties have different genotypes, not all spring wheat would be expected to display salt tolerance. It is necessary to further investigate the mechanism of salinity tolerance in wheat genotypes.

Root hairs are single-cell extension from epidermal cells, which have important function in water/nutrient uptake (He et al., 2015). Development and morphogenesis of root hairs are greatly regulated by many environmental factors, such as nutrition, abiotic and biotic stresses. Recent studies have shown that plants can protect themselves by reducing the length and density of root hair and the absorption area of excessive Na^+^ when they sense stress signals (Wang et al., 2008; Liu et al., 2023). However, the differences in root hair growth between salt-tolerance wheat varieties and salt-sensitive wheat varieties under salt stress are not well understood. Here, the effect of salinity on root hair growth between QM6 (salt-tolerant) and CS (salt-sensitive) was observed via stereomicroscope and SEM. QM6 exhibited significantly more and longer root hairs in the 0.30% NaCl treatment than that in the control (Figures 2A, B), whereas there was a significant reduction in root hair density and length in CS compared with the control (Figures 2B, C). Moreover, QM6 had much more and longer root hairs under salt stress than CS (Figure 2). The results implied that the higher salt tolerance in QM6 could be attributed to its well-developed root hairs.

Salinity can also impair the normal structures and function of the organelles, such as chloroplasts. Chloroplasts are specialized plastids present in plants and algae, and their main function is photosynthesis, through which chloroplasts generate energy essential for plant growth and crop yield (Wang et al., 2024). chloroplasts also play a multitude of crucial roles beyond photosynthesis, engaging in the biosynthesis of amino acids, fatty acids, nucleotides, lipids, and vitamins, as well as the production of phytohormones, starch, and pigments (Neuhaus and Emes, 2000; Li J. Y. et al., 2022). Salinity can affect chloroplast size, number, shape, lamellar organization, starch accumulation and so on (Zahra et al., 2022). Additionally, chloroplasts are major sites for ROS production in plants subjected to salt stress (Wang et al., 2024). Thus, salt stress negatively affects the function of chloroplasts, and further impairs plant growth and development. In wheat, several changes have been associated with chloroplast structure in response to salt stress (Zuo et al., 2020). Chloroplast thylakoid membranes swelled and the accumulation of starch in the chloroplasts increased in wheat cultivars to slightly different extents under high salinity (200 mM NaCl) (Salama et al., 1994). Chloroplasts of salt-sensitive cultivars displayed an increase in volume possibly due to changes in the ionic composition of the stroma. Changes in the ionic composition of starch-degrading enzymes may also be linked with excessive starch deposition (Salama et al., 1994). Indeed, in this study, the chloroplast of QM6 possessed clearly two envelope membranes, a well-developed internal membrane system, composed of grana and long stromal thylakoids (Figure 3A). In contrast, the chloroplast shape of CS was deformed and broken (Figure 3B). Its two envelope membranes were broken and incomplete, and the grana and stoma thylakoids of chloroplast were very irregular (Figure 3B). The most important physiological process that takes place in chloroplasts is photosynthesis, which is also sensitive to salt stress (Wang et al., 2024). The initial impact of salinity on plants is osmotic stress, which leads to stomatal closure. Stomatal closure in turn impacts the process of carbon fixation during photosynthesis by limiting CO2 supply, resulting in decrease photosynthetic activity and photosynthetic rate (Amor et al., 2020). Under salt stress, the degree of photosynthetic efficiency varies differentially in diverse genetic backgrounds, which can be used as indicators to distinguish the salt tolerance of different crop genotypes (Pradhan et al., 2019). In this study, our results showed that QM6 had increased photosynthetic activity as reflected by higher Ci, Tr, Gs and Pn under salt stress conditions compared to CS. These results were consistent with previous studies, where Ci, Tr, Gs and Pn in salt-tolerant wheat varieties showed a higher level than in salt-sensitive wheat varieties (Figure 4). Taken together, chloroplast structure in our study implied that the photosynthetic activity in QM6 might be less damaged than in CS plants under salt stress. Thus, it’s necessary to further use a biparental mapping population created by crossing CS and QM6 to identify the genetic components and genes that control photosynthetic efficiency.

**FIGURE 6:**
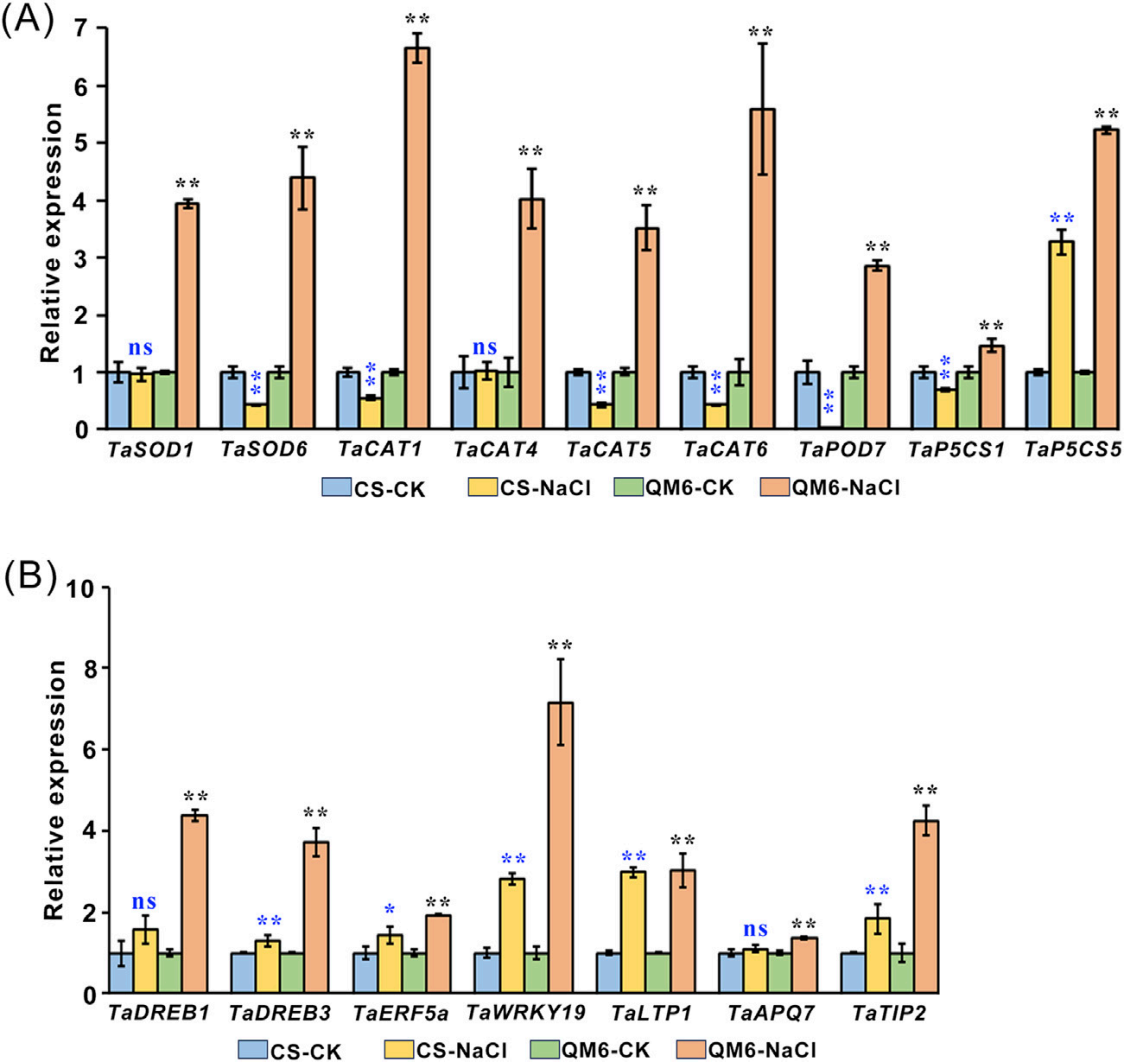
Expression analysis of stress-related genes in the salt-tolerant cultivar (QM6) and salt-sensitive cultivar (CS) under salt stress **(A, B)**. **(A)** ROS-scavenging genes. **(B)** Stress-responsive genes. The relative expression levels of each gene in the leaves of CS and QM6 were normalized with the respective gene set as 1, respectively. *TaActin* was used as the internal standard. All values are presented as mean ± SD. Statistical differences between control and salt treatment groups are indicated by asterisks and were determined using Student’s t-test: ns, not significant; *P < 0.05; **P < 0.01.

Salt stress induces the accumulation of ROS, which have oxidative stress-induced toxic effects on plants. ROS homeostasis is crucial for plant growth, development, and response to salt stress, but the excess production of ROS can cause toxic effects that damage all components of the cell, including proteins, lipids, and DNA, which might be corrected with plant growth and chloroplast structure in our study (Li Z. H. et al., 2022; Mittler, 2002). Detoxification signaling pathways are involved in controlling the homeostasis of cellular ROS levels under salt stress (Yang and Guo, 2018). SOD, CAT, POD and P5CS are ROS-scavenging enzyme and known to be involved in redox homeostasis of cells under various stress conditions (Zhao et al., 2022). In this study, compared to CS plants, QM6 had increased osmotic adjustment capacities and active antioxidant metabolism as reflected by higher contents of soluble sugar and proline, and higher activities of SOD and POD, respectively (Figures 5A–D). The accumulation of H_2_O_2_ and O_2_^−^ in QM6 under salt stress was remarkably lower than those in CS (Figures 5E,F). Besides morpho-physiological study, previous research has established that ROS-scavenging genes and stress-responsive genes are essential in plant stress responses (Hayat et al., 2021; Çelik et al., 2019). overexpression of *TaPRX-2A* encoding peroxidase (PRX) improves the tolerance of wheat against salt stress (Su et al., 2020). *TaSOD2* overexpression in wheat and Arabidopsis enhanced the resistance to salt and oxidative stress (Wang et al., 2016). Multiple wheat transcription factors have been identified as playing pivotal roles in enhancing plant tolerance to drought stress (Manna et al., 2021). For instance, the wheat DREB TF TaDTG6-B positively regulates *TaPIF1* transcription to enhance drought tolerance in wheat (Mei et al., 2022). *WRKY2* and *WRKY19* positively contribute to plant tolerance to drought, salt, and cold stresses in transgenic Arabidopsis (Niu et al., 2012; Gao et al., 2018). Other stress-responsive genes, such as *ERF5a* and *TIP2*, are involved in plant response to drought and salt stress (Eini et al., 2013; Wang et al., 2011). To elucidate how QM6 promotes root and shoot growth under salt stress compared with CS, the expression patterns of some salt stress marker genes including ROS-scavenging genes (*SOD*, *CAT*, *POD* and *P5CS*) and stress-responsive genes (*TaDREB1*, *TaDREB3*, *TaERF5a*, *TaWRKY19*, *TaLTP1*, *TaAQP7*, and *TaTIP2*). The results showed that the ROS-scavenging genes including *TaSOD1/6*, *TaCAT1/4/5/6*, *TaPOD7*, and *TaP5CS1/5* were upregulated in QM6 plants compared to the control under salt stress conditions. In contrast, *TaSOD6*, *TaCAT1/5/6*, *TaPOD7*, *TaP5CS1* were dramatically downregulated in CS plants compared to the control under salt stress conditions. Furthermore, the *TaP5CS5* gene expression were significantly enhanced in QM6 plants compared with CS (Figure 6A). Although the *TaDREB3*, *TaERF5a*, *TaWRKY19*, *TaLTP1*, and *TaTIP2* gene expressions were all upregulated in both QM6 and CS under salt stress conditions, the change in the degree of these upregulated genes in QM6 was significantly higher than that in CS (Figure 6B). Therefore, our results indicate that QM6 had stronger adaptability to salt stress than CS probably *via* reducing ROS accumulation and inducing the expression of the downstream stress-related genes involved in ROS scavenging and defense mechanisms. Thus, these differentially expressed genes between CS and QM6 are likely to be candidate genes for salt tolerance, and can be developed as new diagnostic markers for marker-assisted selection in breeding programs in the future research. The application of molecular markers is productive for different genetic studies, molecular marker-assisted selection, and screening of candidate genotypes for stress tolerance. It has been used in several previous studies and reported to be very informative and highly capable to distinguish between wheat genotypes for salinity tolerance. (Shahzad et al., 2012; Elshafei et al., 2019; Hasanuzzaman et al., 2022; Al-Ashkar et al., 2020). Collectively, given that accurate phenotyping is crucial for identifying potential salt-tolerant wheat candidates, a combined approach of phenomics and genomics is vital to guarantee the successful cultivation of wheat varieties that exhibit tolerance to salinity. The tolerant and moderately tolerant genotypes have been identified as resource base population could to be utilized suitably for further improvement programme for salt tolerance in wheat.

## 5 Conclusion

In this study, morphological, structure, gene expression comparisons of the salt-sensitive wheat cultivar (CS) and salt-tolerant cultivar (QM6) were carried out under different salt stress levels. We have found that QM6 performed better in root and shoot growth, root hairs, chloroplast structure, and physiology in response to salinity compared with CS. Moreover, the expression level of ROS-scavenging genes and stress-responsive genes displayed more transcripts in QM6 than that in CS. Better understanding about the morphological, structural, physiological, biochemical, and molecular mechanisms of wheat varieties in response to salinity may enable further improvement in salt tolerance of wheat, and development of more salt-tolerant wheat cultivars.

## Data Availability

The original contributions presented in the study are included in the article/Supplementary Material, further inquiries can be directed to the corresponding authors.
