# Supplementary material for: Effects of salinity stress on morphological structure, physiology, and mRNA expression in different wheat (Triticum aestivum L.) cultivars
Source: Front Genet. 2025 May 30;16:1535610. doi: 10.3389/fgene.2025.1535610 (PMC12162971; doi:10.3389/fgene.2025.1535610)
Supplement: Supplementary file 1 [file Table1.doc]

**Table S1. | Primer sequences used for qRT-PCR in this study**

| Primer Name | Left Primer ( 5’- 3’) | Right Primer ( 5’- 3’) |
| --- | --- | --- |
| *TaActin* | TATGCCAGCGGTCGAACAAC | GGAACAGCACCTCAGGGCAC |
| *TaSOD1* | GACGCTGATGATCTTGGCAAGG | ATCTTAGCCCTGGAGCCCGATG |
| *TaSOD6* | GGTGGGCATGAGCTCAGCCTCA | CCAGGTAAAACGAGAATGGCGT |
| *TaCAT1* | GCGAGAAGATGGTGATCGA | AGGAGAGCCAGATGGCCTTG |
| *TaCAT4* | GGAGAAGACGAGGATCAAGAAG | ACTTGGAGAGGAAGTCGATC |
| *TaCAT5* | CCAGTGGCTCACCCGCCTCGGT | ACACCAACTATCATTGTTCATC |
| *TaCAT6* | GGGCAGAAGCTGGCGTCGCGG | TTCATGGCTACACCCACAGAG |
| *TaPOD7* | CAAGTCACCA CCACTCCAAT | CCAGAGCCACGAGCACCAC |
| *TaDREB1* | AGACCGAGGCGAGAGGAGAT | GCAACCGAATCAGGACCAGTG |
| *TaDREB3* | CTCGATTCGCTTGCTCCTCAG | TCCTGATGACAAGCTGTAGTGTGC |
| *TaERF5a* | CTACACATCCGTCTTCGTTCC | CATCGCACCATTCAGCAGAA |
| *TaWRKY19* | AGGGAAGCATACGCATGACGTGC | GGCGAGATCGTTCAGAATGGCTGT |
| *TaLTP1* | GCCCTCCACCACAACTACAG | CCGGCCAATCTCCTAACTCC |
| *TaAPQ7* | GGCCGGACTGAAGTGTAGAT | ACAGGACAAAGGTGTGGGAT |
| *TaTIP2* | CCCCTACACACGGGCTTTC | TTGGTGAAGTCGCCGCTG |
